# Enhancing Foreign Language Learning Approaches to Promote Healthy Aging: A Systematic Review

**DOI:** 10.1007/s10936-024-10088-3

**Published:** 2024-05-17

**Authors:** Blanka Klimova, Cecília de Paula Nascimento e Silva

**Affiliations:** 1 Faculty of Informatics and Management, Department of Applied Linguistics, University of Hradec Kralove, Rokitanskeho 62, 500 03 Hradec Kralove, Czech Republic; 2 Department of Psychology, Institute of Education, The Federal University of Mato Grosso, Fernando Corrêa da Costa Avenue 2367, Cuiabá, Brazil

**Keywords:** Elderly, Approaches, Foreign language, Review

## Abstract

The main purpose of this study is to investigate the best approaches to teaching a foreign language to older people to help them achieve the desired results and explore their benefits. This review strictly follows the PRISMA methodology for systematic reviews and meta-analyses to identify the core experimental studies that deal with the topic of foreign language learning approaches among the older generations. Altogether eight studies detected were included in the systematic review. The available sources were found in Web of Science and Scopus. The findings indicate that foreign language learning can promote seniors’ welfare and successful aging despite their learning outcomes, which means that the key benefit for third-age foreign language learners while learning a foreign language is not the achieved proficiency level, but the feeling of subjective satisfaction. This can be a good incentive to achieve better learning outcomes, provided that learners have been offered a pleasant and safe learning environment, using suitable learning approaches during which they can build on their acquired knowledge and experience, as well as discuss the topics they are interested in. Thus, foreign language teachers play an important part in seniors’ educational process because their teaching methods and materials when adapted to the elderly’s educational needs can have a positive impact on the maintenance and possibly, enhancement of the older people's cognitive functions and on the improvement of their mental activity, which consequently maintains their healthy aging.

## Introduction

There is a notable growth in life expectancy all around the world and by 2050 the number of the population aged 60 years and more will increase to 2.1 billion according to the World Health Organization (World Health Organisation, 2024). Due to the increasing number of older adults, learning at a later age is a matter that needs to be discussed even more nowadays. Therefore, research on active and healthy aging can have an impact on the lives of many.

The importance of understanding the cognitive changes that normally happen at a later age is essential for differing what is normal, exceptional, and pathological when talking about the intellectual potential of the elderly in society (Burke & Mackay, 2022; Zihl & Reppermund, 2023). Furthermore, research conducted in care homes in Sri Lanka (Wijesiri et al., 2019) showed that there was a high rate of loneliness and depression among healthy older individuals and the feeling of abandonment was the lead component of their emotional suffering. The staff often encouraged the persons to engage in social activities. In agreement, Ware et al. (2017) indicate that both loneliness and depression hurt one’s cognitive functioning and that involvement in leisure activities can decrease the risk of dementia. Educational interventions seem to allow older adults to stimulate cultural and social practices leading to brain activity and health outcomes (Hong et al., 2023; Sibai & Hachem, 2021). Thus, lifelong learning courses can potentially work as a useful approach to health promotion once they reduce health and social inequity, helping older adults maintain a good quality of life and sustain their later life development and personal autonomy inside their communities (Bar-Tur, 2021; Narushima et al., 2018).

The impact of language learning on the aging brain is crucial for understanding experience-dependent neuroplasticity and the neural mechanisms underlying language learning, even though the cognitive consequences of language learning may be limited to language acquisition itself. According to Chang and Chou (2015), despite their long experience with the learning process, elderly individuals struggle with physical, mental, and cognitive development decline. Therefore, the needs behind their desire to learn a new language are essential to keep them motivated to learn. Moreover, bilingualism has an important role in fluid mental performance when used to adapt to constant changes while processing information and it may heighten cognitive functions in healthy older individuals (Klimova, 2018). In agreement, research shows that foreign language learning (FLL) has great benefits for the enhancement of cognitive functions, such as working memory, and promotes many other aspects of life, e.g., traveling and communicating (Klimova, 2021). Hippocampal volume and associative memory performance evidence of age-related declines imply that these characteristics may be major limiting factors for second language learning in older persons (Van Petten, 2004). In the same context, it has been suggested that the hippocampus is essential for associative memory, which is particularly relevant to language learning. Therefore, it was also thought to be a key predictor (Duff & Brown-Schmidt, 2012). Research (Kacetl & Klimova, 2021) also indicates that teaching a foreign language among the older generations should focus on the student and communicative methods, especially with topics related to their daily life and listening comprehension. It also suggests that three main areas should be taken into consideration when teaching seniors: the learning environment, the teaching style, and the establishment of a teacher-student rapport. Furthermore, the most recent study protocol by Makri et al. (2023) suggests that learning English, especially through learning English songs, could enhance the cognitive abilities, psychological symptoms, and well-being of people even with Mild Cognitive Impairments and thus avoid the deterioration of their deficits that may lead to dementia.

The matter of time (age) in L2 learning is essential when different and contrasted viewpoints are mentioned. It is well recognized that the time of second language acquisition is crucial, with the average result being a better level of proficiency acquired if the language is learned during infancy as opposed to maturity (Johnson & Newport, 1989). While complete second-language perfection is less likely to be attained by an adult student (Nilsson et al., 2021), advanced foreign language proficiency is nevertheless possible in this age group (Hartshorne et al., 2018; Wang et al., 2023). It has also been suggested that learning a foreign language can help older persons' cognition by activating their vast linguistic network and possibly slowing age-related cognitive decline (Antoniou et al., 2013). However, the evidence supporting the general cognitive benefits of language learning in older adults has been weak (Ramos et al., 2017; Ware et al., 2017), and it has been just recently demonstrated that an introductory Italian course did not confer any general cognitive advantage compared to relaxation training (Berggren et al., 2020). Independent of attained vocabulary competency, the study of Nilsson et al. (2021) showed no proof of differential structural change after language instruction. Nevertheless, it was discovered that hippocampus volume and associative memory capacity before the intervention were reliable predictors of vocabulary competency after the language course. The findings imply that vocabulary learning in older age benefits from increased hippocampus volume and improved associative memory ability, but that the very beginning of foreign language learning does not result in discernible changes in brain morphology in old age. This is in line with another study by Fong et al. (2022) whose findings also confirm that semantic and episodic memory functions in vocabulary learning in older learners play important roles.

Learning approaches represent another important constituent in the process of language learning for elderly people (Bosisio, 2019; Kacetl & Klimova, 2021). For instance, Mora et al.’s (2018) study aims to identify the language learning approaches of a group of seniors who took an English course in Cuenca and to ascertain the degree to which these approaches are affected by socio-demographic factors like age, prior English proficiency, educational attainment, and employment before retirement. The findings show that elderly people employ all of Oxford’s categorical approaches (Oxford, 1990), primarily the metacognitive ones, which entail reflecting on, organizing, evaluating, and monitoring one's learning process. Additionally, the findings show a positive link between factors, such as age, English proficiency, education level, and occupation.

Therefore, this research review study intends to explore the best approaches to teaching a foreign language to older people to help them achieve the desired results. This is a unique review study with a focus on foreign language teaching among the older generation, as there are very few studies that have addressed this topic and contributed to reducing the social and economic burden on the state caused by the growth of this target group (cf. Bialystok, 2017). The research questions to provide responses to the set aim are as follows:

- *What are the benefits and pitfalls of learning a foreign language at a later age?*
- *What are the best approaches the foreign language teachers should use to help older people learn a foreign language?*
- *What are the pedagogical implications of teaching a foreign language to third-age learners?*

## Methodology

The following systematic review strictly follows the PRISMA methodology for systematic reviews and meta-analyses to identify the core (principal, essential) experimental studies that deal with the topic of foreign language learning approaches among the older generation. All other studies, such as theoretical studies, conceptual studies, or survey studies were excluded. Only peer-reviewed journal articles that appear in Scopus and Web of Science were considered. No time frame was applied, i.e., all relevant studies in the two databases were included without any time limitation. The search period finished on 31 December 2023. Only open-access articles written in English were considered, however, the L2 did not need to be in English. Grey literature was excluded as well as it does not guarantee a rigorous review process and it cannot be considered reliable enough to be included in this study.

## Inclusion Criteria

- Empirical and experimental studies dealing with the research topic, i.e., foreign language learning approaches among the older generation, such as Fong et al. (2022) or Nilsson et al. (2021).
- Peer-reviewed journal articles.
- Scopus and Web of Science databases.
- Studies written in English.
- Open-access articles.

## Exclusion Criteria

- Non-experimental studies, such as theoretical studies, or conceptual studies were excluded.
- Grey literature, such as conference proceedings.
- Other databases than Scopus or Web of Science.
- Studies written in other languages than English.

### Search String

(“older people” OR “elderly”) AND (“language learning” OR “foreign language*” OR “language acquisition”) AND (“learning approaches” OR “learning techniques”).

After all search criteria were applied, the search generated 73 studies altogether that were later analyzed for their relevance to the topic. Referential backtracking was also conducted to verify if any relevant studies could have been omitted from the search. The final number of the studies yielded was nine to be included in this systematic review.

## Results

Altogether nine studies were detected. Four articles of the eight selected originated in the Czech Republic (Klimova et al., 2021a, 2021b; Pikhart & Klimova, 2020; Pikhart et al., 2021). The five remaining studies are from Austria, Germany, Ukraine, and the USA (Grossmann et al., 2023; Grossmann et al., 2021; Hertzog et al., 2020; Pfenninger & Kliesch, 2023; Sandal et al., 2019). The key topics of these studies were the motives behind seniors’ interest in acquiring a second language at a later age as well as the effects of FLL on the quality of life of older people. One of the articles investigated self-regulatory behavior and its effect during the learning period (Hertzog et al., 2020). The study by Pfenninger and Kliesch (2023) examined L2 variability and its impact on L2 acquisition among older adults. The number of participants in the sample ranged from 60 to 205 participants. Most studies (Grossmann et al., 2021; Klimova et al., 2021a, 2021b; Pfenninger & Kliesch, 2023; Pikhart & Klimova, 2020; Pikhart et al., 2021) used experimental groups, except for two studies (Grossmann et al., 2023; Hertzog et al., 2020; Sandal et al., 2019), which conducted controlled trials. In addition, three articles compared experimental groups between the Czech Republic and Poland (Klimova et al., 2021a, 2021b; Pikhart et al., 2021). The outcome measures usually involved questionnaire surveys, statistical analysis, and study trials. Some studies also used additional methods, such as interviews to identify subjective factors and motives for L2 acquisition in elderly people.

The results indicate that FLL can promote seniors’ welfare and successful aging despite their learning outcomes. Moreover, one of the studies (Hertzog et al., 2020) showed that older adults manifested a lower region of proximal learning due to their lack of belief in their ability to learn. Furthermore, according to Klimova et al. (2021a) and Pikhart et al., (2021), there is a correlation between the level of education, age, the sexes, and the number of languages studied during the response to the perceived effects of a foreign language learning impact.

As far as the pedagogical implications are concerned, the findings revealed that teachers should improve the students’ cognitive behavior and reserve by combining exercises and using different approaches to fit the students’ characteristics and traits, such as scaffolding and performing feedback when necessary. For instance, Grossman et al., (2023) propose the use of technologies for learning grammatical rules and vocabulary, as well as individually guided and planned by a specialized teacher, which would better allow meeting participants at their performance level. Thus, educators play an important part in FLL because their teaching methods and materials when adapted to the elderly’s educational needs can have a positive impact on the maintenance and possibly, enhancement of the older people's cognitive functions and on the improvement of their mental activity, which consequently maintains their healthy and happy aging.

Table 1 below provides a summary of the key findings on the research topic according to the recent date of the publication.

**Table 1:** An overview of the main findings from the detected studies

Study and country of origin	The objective of the study	Research sample and procedure description	Instruments	Findings	Pedagogical implications
Grossmann et al., (2023) Germany	To examine whether FLL can improve executive attention and executive functions in healthy older adults	34 German-speaking monolinguals between the ages of 65 and 80 were assigned to a language learning or a waiting list control group. The language learning group was studying Spanish five days a week for 1.5 h for a total of 3 weeks. The waiting list control group received no intervention	Language and social background questionnaire, comprehensive computer-based test battery, cognitive reserve index questionnaire, stroop interference test, divided attention, a subtest of the perception and attention function battery, and many other tasks on secondary outcomes	The findings show that learning a foreign language does not generally foster executive attention or executive functioning. However, elderly with poorer baseline cognition may benefit cognitively from foreign language learning in response inhibition	FLL should meet learners’ abilities and needs. The authors also suggest that technology-assisted learning of grammatical rules and vocabulary might be a solution, as well as individually guided and planned by a specialized teacher, would better allow meeting participants at their performance level
Pfenninger and Kliesch (2023) Austria	To investigate whether different types of intraindividual variation in L2 performance and cognitive functioning are related, and how and when they influence L2 development longitudinally in older adulthood	26 German-speaking adults aged 62–79 who were taught L2 English for 2 × 90 min per week over 6 months	Mixed-methods design (questionnaire, cognitive test battery, L2 tasks, Socio-affective assessment	The results reveal that L2 variability non-linearly affects L2 growth, being especially large during periods of rapid development	Older people are particularly motivated by a social stimulus, being a part of a group, to learn a FL. Based on the findings on the L2 variability, teachers should consider the individual characteristics and needs of their older students
Grossmann et al., (2021) Germany	To evaluate the effects of short and intensive foreign language learning on executive functions in healthy older adults	60 native German-speaking monolingual healthy older adults, aged 65–80 years; two trial arms: Language Learning Group (LLG) and waiting list control group (WLCG). The LLG group will attend a face-to-face Spanish course for beginners for 90 min from Monday to Friday for 3 weeks	Baseline Variables, Vienna Test System (VTS), Cognitive Reserve Index Questionnaire (CRIq), Language and Social Background Questionnaire (LSBQ), Cognitive Functions Dementia test (CFD), Flinders Handedness survey (FLANDERS), Stroop Interference Test (STROOP), Divided Attention (WAFG), Statistical Analysis	Learning a foreign language could promote healthy cognitive aging and prevent cognitive decline in older adults	FLL can promote cognitive reserve which is attached to mental activity, thus it benefits cognitive functions
Klimova et al., (2021a) Czech Republic	To investigate the motives to learn a foreign language at a later age and the different aspects of well-being that stimulate L2 acquisition for healthy older people	Two experimental groups, one from the Czech Republic with 105 respondents and a second experimental and comparative group from Poland with 100 respondents. Both groups of learners aged 55 years and more	Questionnaire survey, Statistical Analysis, t-Student test, and ANOVA test with post-hoc, the Spearman coefficient	The results indicate that seniors’ positive learning outcomes and overall satisfaction are to some extent high when attending foreign language classes at an older age	Teachers should take into consideration the student’s characteristics and traits and teach using, for example, drilling exercises, scaffolding, and providing materials in a visible font
Klimova et al., (2021b) Czech Republic	To explore the positive effects that foreign language learning (FLL) might have on healthy older individuals	Two experimental groups; participants aged from 55 to 80 or more years old, one from the Czech Republic, 92 participants, and another from Poland 100 participants	Questionnaire Survey, Statistical Analysis, and Research Questions	The results revealed four significant factors involving the positive effects of learning a foreign language. Therefore, it showed that FLL improves seniors’ subjective welfare and successful aging	Learning a foreign language is a non-pharmacological method to maintain healthy and happy aging
Pikhart et al., (2021) Czech Republic	To evaluate and compare the positive effects of L2 acquisition in two different regions	Two groups of older individuals from Poland and the Czech Republic, aged 55 + years old. The Czech sample consisted of 105 participants and the polish sample comprised 100 participants	Questionnaire survey	A senior’s quality of life might be significantly improved by learning a second language	FLL can work as a psychosocial rehabilitation tool since it maintains healthy aging
Pikhart and Klimova (2020) Czech Republic	To investigate the level of satisfaction when taking a L2 course and its reflection on successful and active aging	The experimental group consisted of 105 respondents from the Czech Republic with 55 and 80 + years old. Two control groups; the first one consisted of 102 young adults, aged between 19 and 23 years. The second control group consisted of 102 subjects older than 55 years, similar in age to the experimental group	Questionnaire survey	The findings showed that FLL improved the older participants’ welfare, despite their learning outcomes	The L2 acquisition can improve a senior’s quality of life
Sandal et al., (2019) Ukraine	To examine the motives by which older people start to learn English as well as their educational needs	Experimental research was conducted with 64 active older adults, aged 60 to 89 years old, who were the students of the centre of education and social activity for elderly people “second youth”	Online test, Statistical analysis, survey method, interviews, and questioning	The results show the importance of different practices when teaching older adults to increase educational efficiency	Teaching materials and methods should be adapted to the elderly’s educational needs

## Discussion

Many people believe that learning new languages is best done when they are young and that it gets tougher as they get older. The ability of the brain to create and reorganize synaptic connections, known as neuroplasticity, is the basis of this idea. Although it is true that as one gets older, this ability declines, many researchers today hold the opinion that learning a foreign language at an older age may have other benefits that contribute to the overall well-being of older individuals (Klimova & Pikhart, 2020). The discussion below responds to the research questions set in the Introductory part.

The findings of this review indicate that learning a foreign language can bring several benefits to older people, such as subjective satisfaction (Klimova et al., 2021a, 2021b; Pfenninger & Kliesch, 2023; Pikhart & Klimova, 2020; Pikhart et al., 2021), enhanced cognitive skills (Grossmann et al., 2023; Grossmann et al., 2021), and motivation why to study a foreign language (Pfenninger & Kliesch, 2023; Sandal et al., 2019). Similar findings were confirmed by other research studies. For example, Klimova (2018) in their study explains that bilingualism plays an important role in delaying cognitive decline and supports it with findings from the experimental studies by Bialystok et al. (2007) or Kroll and Dussias (2017). However, the main incentive why older people study a foreign language is not their desire to achieve excellent results, but the incentive to share their acquired knowledge and experience with peers of the same age and simply, engage in socializing with them (cf. Klimova et al., 2021a, 2021b; Pfenninger & Kliesch, 2023). On the contrary, the findings of this review indicate several drawbacks which hinder foreign language learning among older individuals. The results (Hertzog et al., 2020; Sandal et al., 2019) show that older people at a later age are not able to reach a high level of a foreign language. This is due to several reasons, e.g., physical impairments (problems with hearing, eyesight, or movement), language training being less effective than relaxation training (Berggren et al., 2020), as well as lower self-esteem, or short-term memory (cf. Antoniou et al., 2013).

As far as the teaching approaches are concerned, Sandal et al. (2019) suggest that teaching materials and methods should be adapted to older learners’ needs. This was confirmed also by other research studies in this review, such as Grossman et al., (2023). Generally, doing the needs analysis in foreign language classes is the first step that helps identify the learners’ needs, desires, prerequisites, and learners’ language background and thus, ensures successful learning outcomes (cf. Axmedovna et al., 2019). Klimova et al. (2021a) expand that teachers should consider their personalities and learning preferences. Furthermore, the authors report that older people need more time to do tasks and remember individual language structures, words, or phrases. Thus, more drilling exercises and scaffolding should be employed while teaching them. This is true not only for teaching individual language skills, such as reading, writing, listening, and speaking but also for instructions. Older people usually welcome to have instructions in their native language (Klimova & Sanda, 2021). In addition, due to their physical impairments, learning materials also should not be dense and written in small font (cf. Klimova & Sanda, 2021). Research also suggests that rather than introducing a great amount of new information and learning techniques, it may be more beneficial to stimulate older learners to retrieve and rely on previously acquired knowledge and consolidated learning approaches (Bosisio, 2019). More recently, research has shown that younger older people also tend to use technologies when learning a foreign language (Olson et al., 2011; Yap et al., 2022), which can enhance their learning in informal settings, as well as connect them with their peers online. According to Mora et al. (2018), the main language learning approaches of older people in learning a foreign language are primarily metacognitive ones, which are related to reflecting on, organizing, evaluating, and monitoring one’s own learning process. Teachers should also recognize elderly people’s efforts and successes in learning a foreign language, and provide them with positive feedback to boost their confidence and motivation (Seven, 2020; Thohir, 2017). Furthermore, the findings of this review study also evaluate that teaching methods should be focused on the student’s needs (Klimova et al., 2021a). Therefore, classroom materials should be used and made considering, for instance, seniors’ visual or hearing impairments and if necessary provide a slideshow presentation with a visible font. Moreover, not only physical characteristics and traits should draw the teachers’ attention, but also the cognitive aspects of each individual to best approach their cognitive strengths.

Thus, the pedagogical implications for third-age learners involve considering their unique characteristics, needs, and motivations. By prioritizing relevance, adapting to cognitive changes, focusing on practical skills, creating a supportive environment, promoting self-directed learning, incorporating multimodal approaches, assessing progress, as well as offering them variability in their tasks, teachers can effectively facilitate language learning for third-age learners and enhance their overall learning experience (cf. Kacetl & Klimova, 2021).

This research is limited in some aspects. Most of the articles are studies conducted in Europe, except for one (Hertzog et al., 2020) which was conducted in the United States. Moreover, this study does not discuss the differences between the learning outcomes of teaching in a classroom or teaching individually in a private lesson. Future research could investigate the same aspects but in other parts of the world. It also could investigate the benefits of both methods as well as their weaknesses to help seniors choose the option that better fits their needs. However, this research deliberated why a classroom environment is important when it comes to the promotion of social interaction, especially because most elderly people suffer from abandonment and loneliness. Thus, FLL contributes to a senior’s quality of life.

## Conclusion

In conclusion, the findings of this study indicate that the key benefits for third-age foreign language learners while learning a foreign language is not the achieved proficiency level in language learning, but the feeling of subjective satisfaction, which, on the other hand, can be a good incentive to achieve better learning outcomes. However, learners have to be offered a pleasant, supportive, inclusive, and safe learning environment, as well as suitable learning approaches during which they can build on their acquired knowledge and experience, as well as discuss the topics they are interested in. As Grossman et al., (2023) emphasize FLL should be promoted since it contributes to the strengthening of cognitive reserve and as a non-pharmacological approach, it does not do any harm to the elderly.

Therefore, future research should help more understand how learning a foreign language at an older age could lower the likelihood of cognitive decline, respectively different kinds of dementia, as well as halt brain degradation in seniors who already have these conditions.

## Data Availability

All authors made sure that all data and materials as well as software application or custom code support their published claims and comply with field standards.
